# Predominance of synovial sensory nerve fibers in arthrofibrosis following total knee arthroplasty compared to osteoarthritis of the knee

**DOI:** 10.1186/s13018-016-0359-0

**Published:** 2016-02-17

**Authors:** Franz Xaver Koeck, Miriam Schmitt, Clemens Baier, Hubert Stangl, Johannes Beckmann, Joachim Grifka, Rainer H. Straub

**Affiliations:** MedArtes - Private Orthopaedic Clinic, Regensburger Strasse 13, 93073 Neutraubling, Germany; Laboratory of Experimental Rheumatology and Neuroendocrine Immunology, Department of Internal Medicine I, University Hospital Regensburg, Franz-Josef-Strauss-Allee 11, 93042 Regensburg, Germany; Department of Orthopedic Surgery, University of Regensburg, Kaiser-Karl-V.-Allee 3, 93073 Bad Abbach, Germany; Sportklinik Stuttgart, Taubenheimstrasse 8, 70372 Stuttgart, Germany

**Keywords:** Arthrofibrosis, Knee, Osteoarthritis, Arthroplasty, Nerve fibers

## Abstract

**Background:**

So far, there exists no golden standard for the treatment of arthrofibrosis (AF) following total knee arthroplasty (TKA). Although pain is a hallmark of AF, nociceptive nerve fibers have never been investigated in affected joint tissue.

**Methods:**

A total of 24 patients with osteoarthritis (OA) of the knee (*n* = 12) and post-TKA AF of the knee (*n* = 12) were included. Along evaluation of typical clinical signs and symptoms by using the Knee Society Clinical Rating System (KSS), the Knee Injury and Osteoarthritis Outcome Score (KOOS), and the Western Ontario and McMaster Universities Osteoarthritis Index (WOMAC index), the innervation of joint tissue was studied by semiquantitative immunofluorescence of nerve fibers.

**Results:**

Patients with AF compared to OA had a lower KSS and lower KOOS. In all compartments (anterior, medial, and lateral recesses), the density of synovial sympathetic nerve fibers was significantly higher in OA compared to AF, which was also true for the density of sensory nerve fibers in the medial and lateral recesses. In synovial tissue of the anterior recess of patients with AF compared to OA, the density of nociceptive sensory nerve fibers was significantly higher relative to sympathetic nerve fibers. This was similarly observed in the neighboring infrapatellar fat pad of the knee.

**Conclusions:**

Similar as in many painful musculoskeletal diseases, this study indicates that patients with arthrofibrosis of the knee after TKA demonstrate a preponderance of profibrotic sensory nerve fibers over antifibrotic sympathetic nerve fibers. This could serve as a starting point for AF therapy with specific antifibrotic pain medication or regional anesthetic techniques.

## Background

Arthroplasty is a safe and satisfactory procedure in the therapy of degenerative knee pathologies. Most people experience great benefit with regard to mobility and quality of life even in long-term follow-up after total knee arthroplasty (TKA) [1, 2]. Nevertheless, reduced motion and stiffness can be a frustrating complication for both, the patient and the surgeon. This is often linked to persistent pain and marked functional problems. Since the beginning of the 1990s, stiffness due to an exaggerated scar formation as observed after anterior cruciate ligament reconstruction was called arthrofibrosis of the knee [3]. In the context of TKA, prevalence of arthrofibrosis (AF) ranges between 1.5 and 4.5 % of all TKA operations [4, 5]. Although no consensus as to the treatment of AF exists, four major procedures are used in patients with AF: (1) closed manipulation under anesthesia [6], (2) arthroscopic debridement and lysis [7], (3) open debridement and lysis of scar tissue, and (4) revision arthroplasty [8, 9]. However, it is well known that each kind of manipulation including revision surgery means additional mechanical stress, which is afflicted with a high risk of recurrence of fibrosis [6]. Also, increased psychological distress is supposed to be a risk factor for postoperative stiffness in TKA [10]. While these problems are recognized, the etiology of postoperative AF is not well understood.

There are several important starting points in the etiology of AF. Activation of the fibroblast and myofibroblast is an important aspect with increased production of collagen type VI [11], alpha-smooth muscle protein [12], β-catenin [13], reactive oxygen and nitrogen species (also in macrophages and neutrophils) [14], and bone morphogenic protein type II [15]. The proinflammatory cytokine interleukin (IL)-1β might also play a role since injection of anakinra, the IL-1 receptor antagonist, was effective in an open study in AF patients [16]. Contiguous to fibroblasts, also profibrotic mast cells and T lymphocytes are involved in the scaring process in AF [17, 18]. Under experimental conditions in animals, transforming growth factor beta and vascular endothelial growth factor were found to be associated with fibrosis [19, 20]. In addition, sonographic studies in humans demonstrated increased neovascularization in the synovial tissue and infrapatellar fat pad (Hoffa) [4]. All these factors relevant for a profibrotic process demonstrate a continuous smoldering low-grade inflammation characterized as a new type of synovialitis [13].

Notwithstanding the enormous progress made, it remains unclear why AF is painful and why this may be linked to scar formation. Articular pain is signaled to the central nervous system via peripheral sensory nociceptive nerve fibers, which are equipped with the major neurotransmitter substance P [21].

In patients with TKA-derived AF of the knee (controls: OA patients), this study was initiated to investigate, in parallel, the density of peripheral sensory nociceptive nerve fibers and the density of peripheral sympathetic nerve fibers in synovial tissue of the knee joint. Patients were characterized by classical instruments such as the Knee Society Clinical Rating System (KSS), the Knee Injury and Osteoarthritis Outcome Score (KOOS), and the Western Ontario and McMaster Universities Osteoarthritis Index (WOMAC index) [22–24]. The proliferative process in articular tissue was estimated by cellular density, which was a perfect marker of synovialitis related to AF [13].

## Methods

A total of 24 patients with OA of the knee (*n* = 12) and AF after TKA (*n* = 12) were included. The characteristics of the patients are given in Table 1. The two groups were not different in age, sex, and systemic inflammation measured by the erythrocyte sedimentation rate or C-reactive protein (Table 1).

**Table 1:** Characteristics of patients under study

	Osteoarthritis of the knee	Arthrofibrosis of the knee
Number of patients	12	12
Age, year	69.2 ± 2.4 [59–86]	66.4 ± 2.4 [55–77]
Gender, men/women	3/9 (25/75)	4/8 (33/67)
Indication for operation	12, primary total knee arthroplasty	12, revision after total knee arthroplasty
Affected side, right/left	6/6	7/5
C-reactive protein, mg/l	3.2 ± 0.6 [0.4–7.2]	8.0 ± 2.9 [0.7–36.6]
Erythrocyte sedimentation rate, mm 1st hour	8 ± 2 [2–19]	21 ± 8 [2–76]

Data are given as means ± SEM, percentages in parentheses, and ranges in brackets

The patients with knee OA underwent primary total knee joint replacement surgery, and the patients with AF underwent open revision with debridement and lysis of scar tissue. All patients were informed about the purpose of the study and gave written consent. The study was approved by the Ethical Committee of the University of Regensburg (No. 11-101-0220).

Severity of joint disease was characterized by the KSS, the KOOS, and the WOMAC index according to standard questionnaires [22–24]. The KSS is a simple but objective scoring system to rate the knee and patient’s functional abilities such as walking and stair climbing before and after TKA [22–24]. The KOOS is developed as an extension of the WOMAC index with the purpose of evaluating short-term and long-term symptoms and function in subjects with knee injury and osteoarthritis. It holds five subscales: pain other symptoms, function in daily living, in sport, and recreation, and knee-related quality of life [22–24]. The WOMAC osteoarthritis index assesses pain, stiffness, and physical function in patients with hip and/or knee OA. It consists of 24 items divided into three subscales (pain, stiffness, and physical function) [22–24].

Although knowing that OA and AF might not differ much in expression of joint complaints and signs of inflammation, we studied these two independent patients groups in order to present comparative groups.

The preparation of the tissue for histology was as described [25]. Fat tissue of the infrapatellar fat pad and synovial tissue of the medial, lateral, and anterior recesses of the knee joint were used for histology. Samples intended for the determination of cell density and detection of nerve fibers were fixed, depending on sample size, for 18 to 48 h in phosphate-buffered saline (PBS) containing 3.7 % formaldehyde and then incubated in PBS with 20 % sucrose for 18 to 48 h. Thereafter, the tissue was embedded in Tissue Tek (Tissue Tek, Sakura Finetek, Zoeterwoude, The Netherlands) and quick-frozen floating on liquid nitrogen.

Histological evaluation has been described in an earlier study [26]. Briefly, the frozen tissue samples were cut into 8–9-μm-thick sections, and cell density was evaluated using DAPI (4′,6-diamidino-2-phenylindol) staining. Cellular density in the tissue was determined by counting stained cells in 17 randomly selected high-power fields (400×) and expressed per square millimeter.

**Fig. 1:**
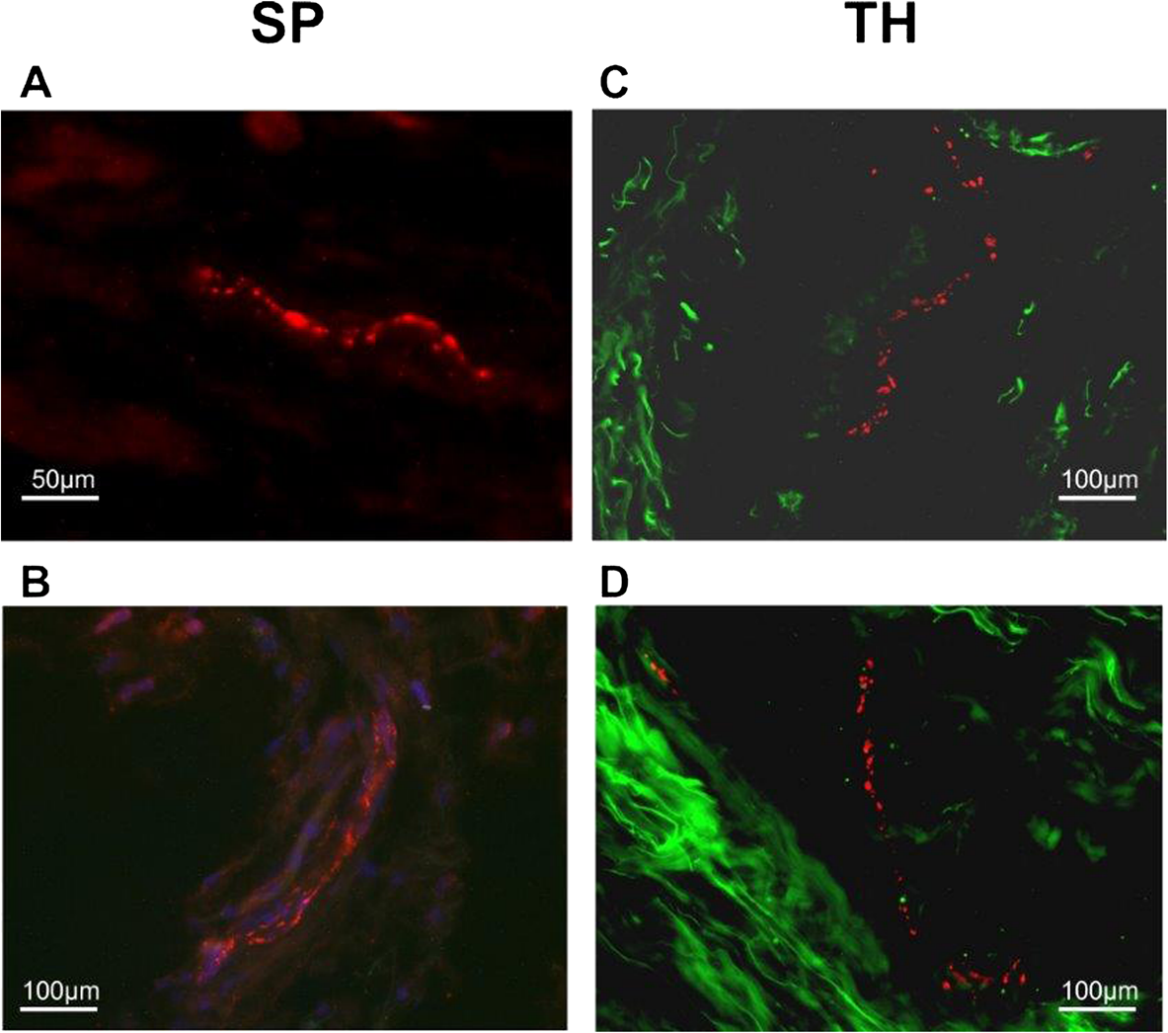
Representative immunohistochemistry of substance P (SP)-positive sensory nerve fibers and sympathetic tyrosine hydroxylase (TH)-positive nerve fibers. **a**, **b** SP-positive sensory nerve fibers embedded in extracellular matrix (**a**) and following a larger synovial tissue artery (**b**; blue fluorescence staining = DAPI, 4′,6-diamidino-2-phenylindol). **c**, **d** TH-positive sympathetic nerve fibers embedded in extracellular matrix (green intrinsic fluorescence demonstrating fibrillar structures). Micrographs were taken at ×400 magnification

The determination of synovial innervation has been described previously [25]. We used a primary antibody against tyrosine hydroxylase (TH^+^, the key enzyme for NE production in sympathetic nerve endings, cat. no. AB152, Chemicon, Temecula, CA, USA) and against substance P (SP^+^, the key neurotransmitter of SP^+^ sensory nerve fibers, cat. no. AB1977, Chemicon). We used an Alexa 546-conjugated secondary antibody (cat. no. A-11010, goat against rabbit IgG, Molecular Probes, Leiden, The Netherlands) to achieve immunofluorescent staining of sympathetic and sensory substance P-positive nerve fibers (Fig. 1).

The numbers of TH^+^ sympathetic and SP^+^ sensory nerve fibers per square millimeter were determined by averaging the number of stained nerve fibers (typical bead chain structure with at least four separated vesicles along the axon, minimum length 50 μm, determined by a micrometer eyepiece) in 17 randomly selected high-power fields of view (400×). We controlled the positive nerve fiber staining by incubating the tissue with polyclonal control antibodies which always yielded a negative result (no red fluorescent staining).

All data are given as mean ± SEM. Box plots give the 10th, 75th, 50th (median), 25th, and 10th percentile. Group medians were compared by the non-parametric Mann-Whitney test (SPSS/PC, Advanced Statistics, V18.0, SPSS Inc., Chicago). *p* < 0.05 was the significance level. Due to the explorative nature of the study, we did not adjust for multiple use of the same data.

## Results

The patients with AF demonstrated a significantly higher Knee Society knee score than the OA patients (Table 2). In addition, the AF compared to the OA patients demonstrated a significantly reduced knee-related quality of life as estimated using the KOOS questionnaire (Table 2). In a form of a trend, pain appeared more severe in the patients with AF compared to OA, however not significantly (Table 2).

**Table 2:** Clinical parameters of patients under study

	Osteoarthritis of the knee	Arthrofibrosis of the knee
Knee Society knee score, points	43 ± 5	33 ± 3*
Knee Society function score, points	52 ± 5	47 ± 6
KOOS pain, points	38 ± 4	28 ± 4**
KOOS other symptoms, points	43 ± 4	33 ± 4
KOOS function in daily living, points	36 ± 4	29 ± 5
KOOS function in sport and recreation, points	7.5 ± 2.8	2.5 ± 1.1
KOOS knee-related quality of life, points	23 ± 3	15 ± 4*
WOMAC pain, points	11 ± 1	13 ± 1
WOMAC stiffness, points	5.0 ± 0.4	5.7 ± 0.4
WOMAC function, points	42 ± 2	47 ± 3
WOMAC total, points	58 ± 4	65 ± 4
Thigh circumference 20 cm above knee, cm	55 ± 3	52 ± 2
Thigh circumference 10 cm above knee, cm	48 ± 3	45 ± 1
Thigh circumference at knee joint, cm	43 ± 1	43 ± 1

Data are given as means ± SEM, percentages in parentheses, and ranges in brackets

*KOOS* Knee Injury and Osteoarthritis Outcome Score

*p < 0.05; **p = 0.072 for comparison of the two groups

**Fig. 2:**
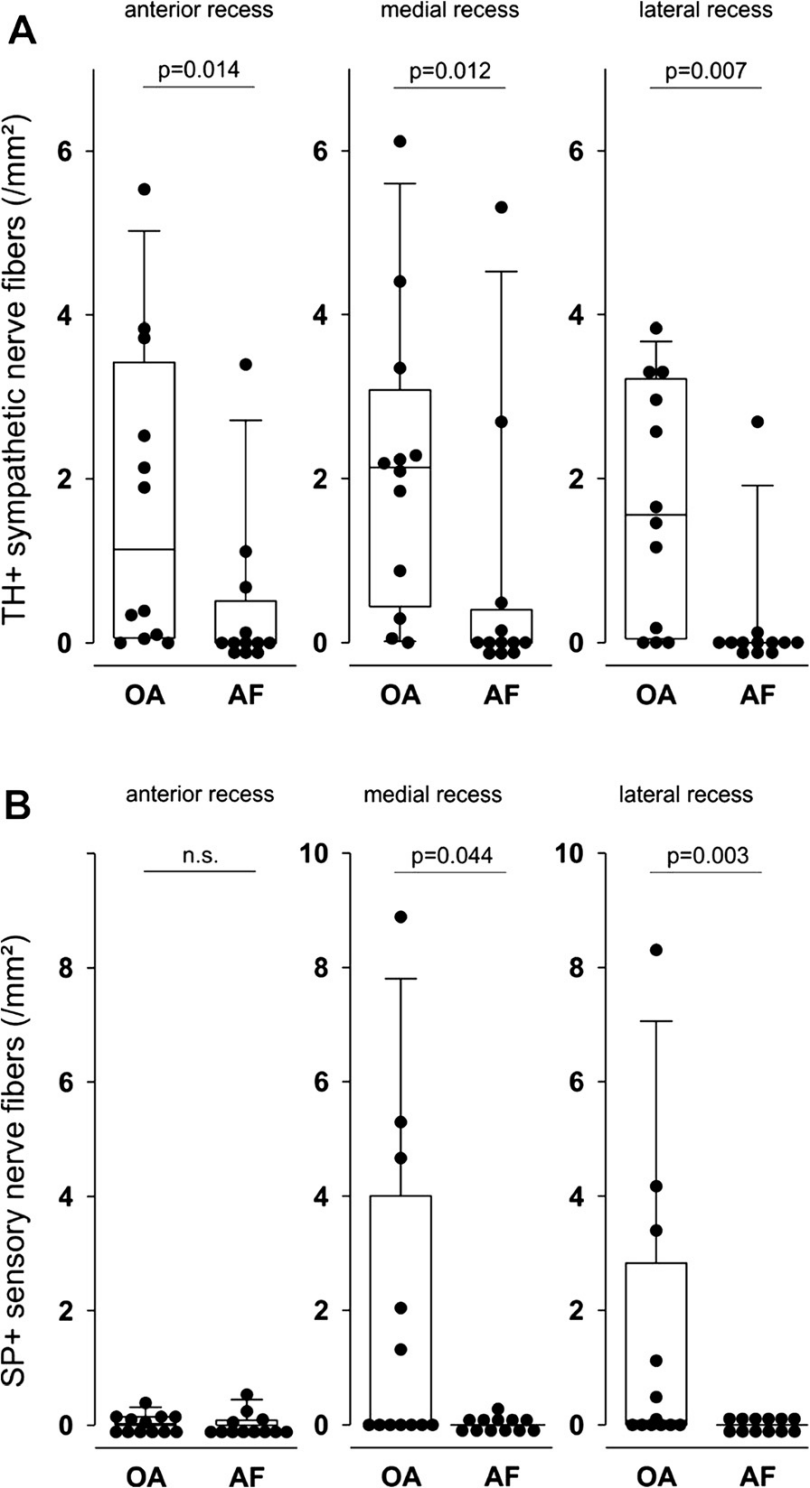
Density of sympathetic tyrosine hydroxylase-positive (TH+) and sensory substance P-positive (SP+) nerve fibers in synovial tissue of three different recesses of the knee in patients with osteoarthritis (OA) and arthrofibrosis (AF). **a** Density of sympathetic nerve fibers. **b** Density of sensory nerve fibers. A *circular symbo*l is the mean value of 17 investigated high-power fields of one patient. *Box plots* give the 10th, 75th, 50th (median), 25th, and 10th percentiles. *n.s.* not significant

In all recesses (anterior, medial, lateral), the synovial density of sympathetic nerve fibers was significantly higher in the OA compared to the AF patients (Fig. 2a). Similarly, in the medial and lateral recesses, the synovial density of substance P-positive sensory nerve fibers was significantly higher in OA compared to AF (Fig. 2b). This indicates that the scaring tissue in AF contains less sympathetic and sensory nerve fibers compared to the OA control group (Fig. 2).

**Fig. 3:**
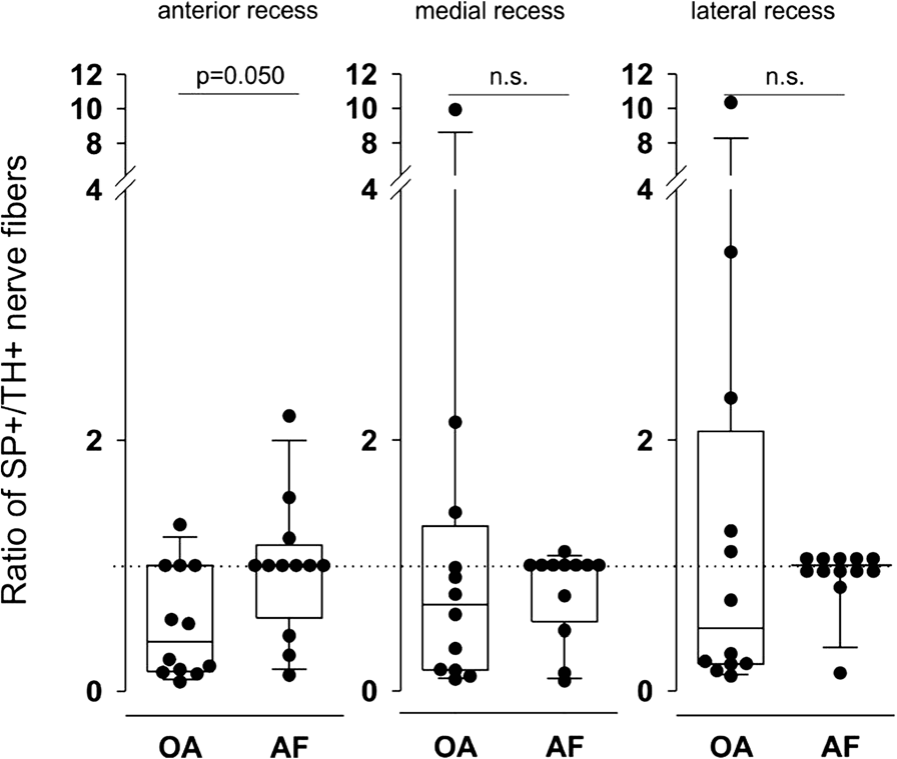
Ratio of density of substance P-positive sensory nerve fibers (SP+) divided by density of tyrosine hydroxylase-positive sympathetic nerve fibers (TH+) in the three recesses of the knee joint. A *circular symbol* is the ratio of one individual patient. *Box plots* are explained in the legend of Fig. 2. *AF* arthrofibrosis, *n.s.* not significant, *OA* osteoarthritis

In order to study a preponderance of a nerve fiber type over the other, ratios were generated in every patient with the density of substance P-positive nerve fibers in the nominator and the density of sympathetic nerve fibers in the denominator [25, 27]. In the anterior recess of the knee, this particular ratio was higher in the patients with AF compared to the patients with OA (Fig. 3). Although a similar trend existed for the medial and lateral recesses of the knee, this did not reach a significant level (Fig. 3).

**Fig. 4:**
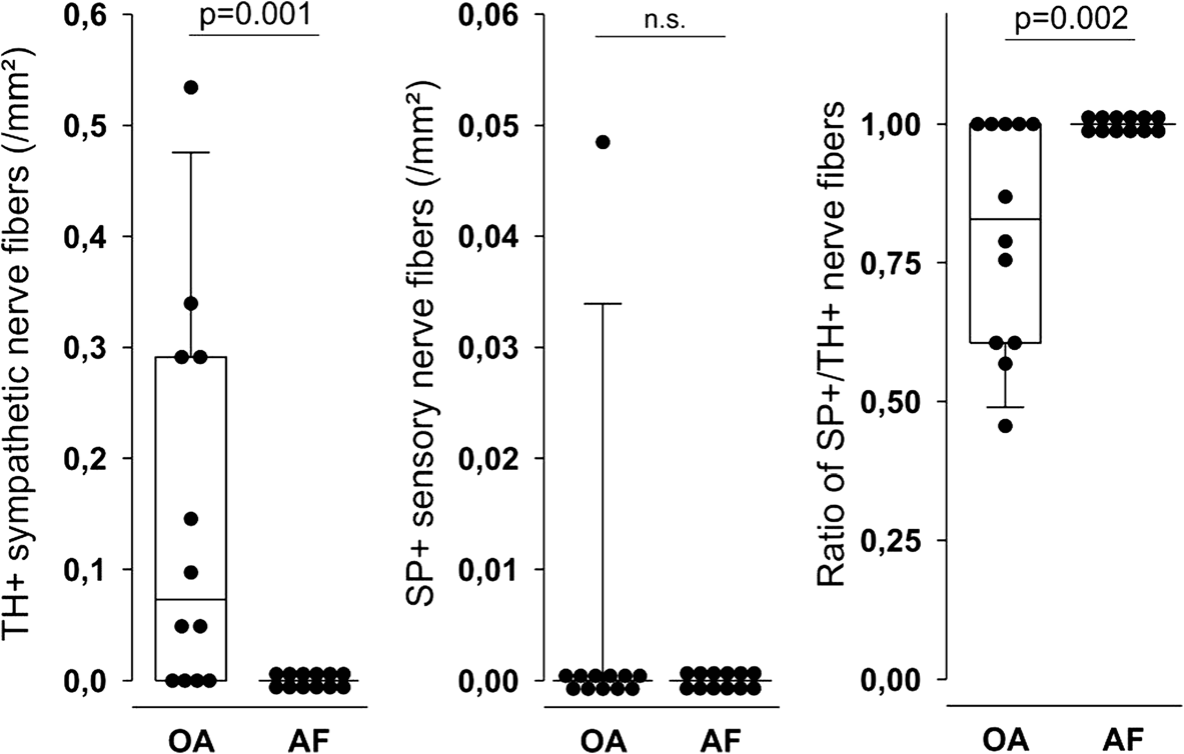
Density of sympathetic tyrosine hydroxylase-positive (TH+) and sensory substance P-positive (SP+) nerve fibers in infrapatellar fat pad in patients with osteoarthritis (OA) and arthrofibrosis (AF). *Box plots* are explained in the legend of Fig. 2. *n.s.* not significant

Since the phenomenon of sensory hyperinnervation in relation to sympathetic innervation was mainly detected in the anterior recess of the knee, additional investigation of the neighboring infrapatellar fat pad (Hoffa) was stimulated. While the density of sympathetic nerve fibers was higher in OA compared to AF (Fig. 4, left panel), the density of substance P-positive sensory nerve fibers was not different between groups (Fig. 4, middle panel). A relative preponderance of sensory over sympathetic nerve fibers in AF compared to OA could be detected when generating the density ratio of substance P-positive divided by sympathetic nerve fibers (Fig. 4, left panel).

## Discussion

To our best knowledge, this is the first study to investigate sensory and sympathetic nerve fiber densities in articular tissue in patients with AF of the knee. The density of sympathetic and sensory nerve fibers was generally higher in OA compared to AF, which probably demonstrates a higher degree of fibrotic scar formation in AF than OA tissue. In addition, in AF compared to OA, in the anterior recess and in the infrapatellar fat pad, the density of sensory nerve fibers was higher in relation to the density of sympathetic nerve fibers. This indicates a preponderance of profibrotic sensory nerve fibers over antifibrotic sympathetic nerve fibers. This phenomenon seems to be located solely in anterior compartments.

Sensory hyperinnervation in relation to sympathetic innervation as a consequence of chronic mechanical or inflammatory stimulation of the tissue has been observed in several diseases: (1) osteonecrosis of the femoral head [28], (2) chronic pruritus and prurigo nodularis [29], (3) Charcot foot [30], (4) Dupuytren’s contracture nodules [31], (5) painful Achilles tendinosis [32], and (6) rheumatoid arthritis [25]. Hyperinnervation can be a consequence of increased production of nerve growth factor which is often observed during inflammation [33]. In AF, early postoperative pain is often related to the ongoing arthrofibrotic process. Thus, we hypothesize that continuous stimulation of pain fibers induces inflammation, and inflammation itself stimulates relative sensory hyperinnervation leading to perpetuation of the process. This might be a possibility for a therapeutical approach of AF in order to perforate this vicious circle. So far, therapeutical strategies focus on closed or open manipulation and mobilization techniques under anesthesia and intense physiotherapy with appropriate pain medication. However, studies report of little success and an increased risk of another revision [34].

Substance P has a strong effect on fibroblast activation and extracellular matrix production leading to increased scar formation [35]. In addition, substance P is a proinflammatory neuropeptide that can aggravate a continuous inflammatory process: substance P stimulates IL-1 and tumor necrosis factor (TNF) from various cell types [36]. We recently demonstrated sprouting of substance P-positive nerve fibers in diseases with exaggerated fibrogenesis such as rheumatoid arthritis, Achilles tendinosis, and Dupuytren’s contracture [25, 31, 32]. Sensory hyperinnervation and substance P might be important factors for aggravation and continuation of AF.

In contrast to substance P, neurotransmitters of the sympathetic nerve endings can have anti-inflammatory effects at high neurotransmitter concentrations: norepinephrine binds preferentially α-adrenoceptors (at high physiological concentrations also to β-adrenoceptors). Adenosine preferentially binds adenosine 1 (A1) receptors (at high physiological concentrations also to adenosine 2 (A2) receptors) [25, 31, 32]. Ligation of β-adrenoceptors or A2 adenosine receptors increase intracellular cyclic adenosine monophosphate (cAMP) levels and ligation of α2-adrenoceptors or A1 adenosine receptors decrease intracellular cAMP levels [25, 31, 32]. Generally, an elevated sympathetic tone due to increased firing rates at sympathetic nerves results in increased levels of norepinephrine and adenosine in the vicinity of the nerve terminal. This leads to an increase of intracellular cAMP in multiple peripheral target cells. Elevation of cAMP by these mechanisms has been repeatedly demonstrated to induce many anti-inflammatory effects on target immune mechanisms such as secretion of TNF or interferon-γ [25, 31, 32]. Thus, the presence of sympathetic nerves at a high fiber density would yield a high enough anti-inflammatory concentration of norepinephrine and adenosine [25, 31, 32]. In addition, sympathetic nerve terminals bear vesicles with endogenous opioids which are able to inhibit release of the proinflammatory substance P from sensory nociceptive nerve fibers [37]. In a highly inflammatory disease such as rheumatoid arthritis, the preponderance of substance P-positive nerve fibers over sympathetic nerve fibers is approximately 8:1, whereas in healthy tissue, the density of sensory versus sympathetic nerve fibers is balanced at 1:1 [25]. Taken together, the presence of sympathetic nerve fibers leading to high concentrations of sympathetic neurotransmitters can inhibit proinflammatory effects of substance P, which might be also relevant in AF. However, the parallel investigation of sensory and sympathetic nerve fibers has never been reported in this TKA complication.

In the scenario of AF, substance P is not a simple bystander but a strong profibrotic factor leading to fibroblast activation and extracellular matrix production [35, 38]. Substance P is a proinflammatory neuropeptide that aggravates a continuous inflammatory process [36, 39]. In the absence of anti-inflammatory catecholaminergic influences, substance P predominance might trigger a vicious circle leading to exaggerated scar formation. It is interesting that this phenomenon only appears in the anterior compartments of the anterior recess and the infrapatellar fat pad. Since the anterior compartments are more prone to mechanical stress, this region is particularly affected.

We are unable to describe the sequence of events and causality in humans because removal of tissue cannot be done in a serial way and manipulations of substance P release cannot be tested. Future studies might address this important aspect in animal models of AF as these models exist in rats and rabbits [19, 20]. In these models, one might block the activity of substance P by specific receptor antagonists, or one might use tachykinin 1-deficient (Tac1−/−) mice (no SP) in order to specify the role of substance P. In the rat model, Watson et al. described that transforming growth factor beta is an outstandingly important stimulus of AF, which nicely fits to the fact that substance P is a perfect stimulator of this cytokine [40]. In addition, transforming growth factor beta stimulates the substance P receptor in a way that the substance P signaling pathway is more active over a longer period of time [41]. Thus, the pain pathway and the fibrosis pathway are tightly coupled. This is also true for the presence of mast cells because substance P sensory nerve fibers are closely linked to mast cells, [34] and mast cells are predominant in fibrotic tissue not tested in the present study [42]. These aspects could serve as a foundation for the development of new drugs interrupting the abovementioned pathways. A limitation of this study might be the relatively small numbers of patients and controls. We also are aware that the ideal control group would be patients without OA which is also known as an inflammatory disease [30, 37]. But as described, this was not feasible due to ethical reasons and the fact that the needed volume of synovial tissue could not be acquired arthroscopically.

## Conclusions

This study indicates that AF patients have hyperinnervation with sensory nerve fibers relative to sympathetic nerve fibers in the anterior compartments of the knee after total knee arthroplasty. Substance P of sensory nerve fibers is probably a critical element in this process because this neuropeptide has profibrotic capacities and stimulates transforming growth factor beta. Future studies in animal models of AF are needed to investigate the role of manipulation of the sensory nervous system.
